# Leveraging the Expertise of the Community: A Case for Expansion of a Peer Workforce in Child, Adolescent, and Family Mental Health

**DOI:** 10.3390/ijerph20115921

**Published:** 2023-05-23

**Authors:** Hillary A. Robertson, Matthew G. Biel, Katherine R. Hayes, Sara Snowden, Latisha Curtis, Dominique Charlot-Swilley, Elyssa S. Clauson, Arrealia Gavins, Caslin M. Sisk, Noel Bravo, Erica E. Coates, Celene E. Domitrovich

**Affiliations:** 1 Department of Psychiatry, Georgetown University Medical Center, Washington, DC 20007, USA; 2 MedStar Georgetown University Hospital, Washington, DC 20007, USA; 3 Milken Institute School of Public Health, George Washington University, Washington, DC 20052, USA; 4 Children’s National Hospital, Washington, DC 20001, USA; 5 Department of Pediatrics, Georgetown University Medical Center, Washington, DC 20007, USA

**Keywords:** family peer support, community health worker, child mental health, workforce

## Abstract

The rise in child and adolescent mental health concerns has led to the need for an expanded workforce to meet the needs of our nation’s families. Peer paraprofessionals (PPs) have proven to be impactful in the areas of adult mental health (MH) and substance use disorders, and for persons with chronic medical conditions. PPs can contribute to addressing child, adolescent, and family MH needs by being deployed in community settings and providing both emotional and tangible support to families and children. Additional use of PPs can address equity gaps in MH services by improving access to support and enhancing the cultural acceptability of MH interventions. A concentrated effort to expand and develop this workforce may help to alleviate the strain on the current MH system. The Georgetown University Infant and Early Childhood Certificate program is a paraprofessional training program that prepares community members to meet the MH needs of families with young children. The authors will describe the results of a qualitative study examining the landscape of peer paraprofessional services in DC that was conducted to support the expansion of the peer workforce to include individuals with expertise in infant and early childhood mental health.

## 1. Introduction

The last decade has seen a well-documented, much-discussed, and still-growing crisis in child and adolescent mental health in the United States [1,2]. Rates of depression, anxiety, loneliness, disruptive behavior problems, and suicide have increased dramatically in young people [3]. These concerning developments preceded the global pandemic and have continued to worsen since 2020 [2,4]. Important demographic shifts in the prevalence of mental health problems in children and adolescents have also emerged, with notable acceleration in rates of depression and suicidal ideation in adolescent girls, as well as marked increases in rates of completed suicide in pre-pubertal children, boys, and Black-identifying youth [2]. These trends have prompted calls for action, including changes in healthcare payment and policy, enhancement of school-based services, development of effective interventions for underserved populations, and strategies to grow the professional mental health workforce [1].

As researchers, policymakers, and clinicians have sought to understand and respond to this ongoing crisis, several issues in pediatric mental health research have emerged as relevant factors to consider. First, disparities in mental health by multiple demographic factors, including race, ethnicity, language, income, gender and sexual identity, and geography, are shaping clinical outcomes for children and adolescents [2]. Racial and ethnic minorities, LGBTQI+(Lesbian, Gay, Bisexual, Transgender, Queer/Questioning, Intersex+) youth, the poor, and rural populations have profoundly inadequate access to effective, evidence-based mental health care [2,5]. Additionally, many interventions with evidence of clinical efficacy were developed and tested with predominantly white populations and very few of these have been adapted with cultural or linguistic specificity for distinct populations. Only a small number of mental health interventions are developed by or with the community they intend to serve and very few leverage culturally specific strengths or protective factors. Importantly, racially marginalized and underserved populations have significantly increased rates of exposure to adversity and trauma, and abundant evidence strongly connects these exposures to childhood-onset mental health problems [2,6]. Finally, there is increasing recognition that family factors, including parental mental illness, parenting skills, and social support for parents, impact the mental health of children and adolescents [7].

While it is critical to address the factors that contribute to the youth mental health crisis, population-level improvements in mental health will not be achieved until the field also addresses the substantial gaps in the mental health workforce. Estimates are that 30,000 child and adolescent psychiatrists are required to meet clinical demand in the United States, while fewer than 7000 are in active practice [8]. Similar shortfalls exist in the fields of clinical psychology, clinical social work, and counseling. Explanations for these gaps include inadequate training opportunities, arduous training regimens, stigma attached to working in mental health fields, and inadequate reimbursement [9].

One strategy with significant potential to expand the pediatric mental health workforce is the training and deployment of community members to provide a range of mental health supports. Such training does not include enrollment in multi-year programs from traditional degree-granting professional schools and universities but instead focuses on briefer vocational instruction. Students in these programs, who have relevant lived experience and cultural familiarity with the population intended to be served, can be considered “peers” to the community members to whom they will provide support. A variety of terms have been used to describe this workforce but for the purposes of the current paper, we will use the term peer paraprofessionals (PPs).

Training PPs to provide support and deliver mental health interventions is a promising strategy for several key reasons. First, this approach is cost-effective, as the educational and training requirements for these roles tend to be significantly smaller than those for degreed clinicians. In addition, research suggests that peer-delivered interventions are effective for addressing a range of physical and mental health conditions. Finally, there is growing research to suggest that within mental health care specifically, there may be unique benefits to PPs providing services [10]. We will argue that within historically divested communities, employing and effectively deploying PPs has the potential to advance equity across both health and economic domains, as this approach may provide effective mental health support to community members while also creating jobs paying reasonable wages and providing entry into the health services workforce. In the context of increasing interest in expanding intervention strategies for child, adolescent, and family mental health care, as well as the ongoing lack of supply of trained clinicians to meet growing demand, the potential contributions of PPs deserves exploration. The goal of this paper is to provide a summary of the research regarding the effectiveness of PPs, to describe our efforts to develop a paraprofessional training program that prepares PPs to meet the mental health needs of families with young children, and to describe the results of a qualitative study examining the current landscape of peer paraprofessional services for families with young children in District of Columbia (DC).

## 2. Effectiveness of Peer Paraprofessional Support

The following sections provide descriptive examples of how PPs have been deployed to address both physical health and mental health and an overview of research regarding the feasibility, acceptability, and effectiveness of these approaches with a particular focus on paraprofessionals who are peers to the individuals they serve.

### 2.1. Adult Physical Health

In health care settings, community health workers (CHWs) are paraprofessionals tasked with providing essential support to individuals living with chronic medical conditions such as HIV/AIDS, diabetes, and chronic kidney disease. These individuals are often part of a patient’s clinical team, working in conjunction with other providers to support management plans and to address contributing lifestyle factors and social determinants of health (SDOH) [11]. Typical CHW roles include chronic disease management and prevention, health education, care coordination, connection to services, social support, community outreach, health promotion, and advocacy. CHWs provide a bridge between health care systems and the community and are deployed within the broader health workforce to improve health outcomes by enhancing quality of care, access to care, health literacy, cultural competency, and attention to SDOH. Through these mechanisms, CHWs can contribute to reducing health disparities, stigma, and health care costs.

According to the American Public Health Association (APHA), a CHW is a “… frontline public health worker who is a trusted member of and/or has an unusually close understanding of the community served” [12]. Having a shared cultural and community background with patients can support management plan adherence and overcome environmental barriers to lifestyle change [13]. This definition highlights the complicated nature of defining the “peer” component of peer support. Membership in a specific geographic, cultural, racial, ethnic, or linguistic community may be one characteristic of a peer. Another definition is shared lived experience with a specific health condition. For example, within HIV/AIDS health services, HIV positive individuals are often recruited to support medication adherence, deliver education, and provide linkage to community resources and emotional support to other HIV positive individuals [14]. In this example, peer status is achieved through shared personal experiences with the health condition and the healthcare system. Thus, CHWs are considered “peers” based upon shared lived experiences based upon cultural, racial, ethnic, linguistic, and/or geographic commonalities with their clients, and they sometimes share lived experiences based upon common health conditions.

A growing body of research suggests that CHWs contribute to improved outcomes for individuals diagnosed with chronic medical conditions. Research on individuals diagnosed with diabetes has found increased knowledge in patients who receive care from CHWs [15]. In a systematic review of CHW effectiveness for the management of diabetes that included eight randomized clinical trials, many studies found a decrease in inappropriate healthcare utilization with the inclusion of CHWs on healthcare teams [15]. Several studies indicate the benefits of CHW-delivered supports on biomarkers as well as psychosocial markers associated with Type One and Type Two diabetes management, with “peer” status defined as having personal experience receiving diabetes care [11,13]. There is research to suggest peer CHW-delivered support for those with chronic kidney disease empowers patients and supports communication between patients and medical staff [16]. In one study of families whose child was diagnosed with asthma, healthcare teams that included CHWs reduced hospitalizations and emergency room visits by over 50% [17]. In addition to providing support, the use of CHWs reduces costs within the healthcare system, especially for low-income or underserved populations [18].

### 2.2. Adult Mental Health

Peer support has also been a vital element of mental and behavioral health care, particularly in the deployment of Peer Support Workers (PSWs) focused on supporting adults with chronic mental illnesses and substance use disorders. In this context, the “peer” status of PSWs is established based upon the paraprofessionals’ own experiences with recovery from a mental health or substance use disorder [19]. Thus, significant overlap exists between CHWs in the realm of physical health conditions and PSWs in the realm of mental health conditions. PSWs in mental health care have been referred to by a range of other terms including peer specialists, peer recovery coaches, peer advocates, and peer recovery support specialists.

Within these roles, individuals act as educators and advocates, promote autonomy and empowerment, and serve as knowledgeable partners who are able to link clients to community support and resources [20]. PSWs also support the development of life skills, communication, and general social functioning [21,22]. For adults with mental health conditions, peer support has been shown to be moderately effective at improving health outcomes and reducing healthcare costs [23,24,25,26]. The supportive role of PSWs compared to the directive role of clinicians has been found to support increased engagement in treatment and life satisfaction [21,27,28]. The well-known role of sponsors within 12-step programs for addiction recovery are often classified as peer support due to the shared lived experience that defines the role [29]. Numerous studies have demonstrated that having a sponsor is associated with increased likelihood of successful abstinence [29,30,31,32,33].

### 2.3. Child and Adolescent Physical Health

While peer support is increasingly available in health systems to address chronic physical and mental health conditions in adults, these services are less common to support pediatric health conditions. For families with children with special healthcare needs, peer support is typically delivered to caregivers by peers with lived experience caring for children with similar health concerns or developmental challenges (e.g., developmental disabilities). This form of support is also known as family peer support or family support services. Family peer support is a vital resource to parents navigating the clinical, social, and emotional support systems available to support children with physical needs and developmental challenges [34]. This form of support can take different forms based on specific needs and circumstances. The most common delivery forms are one-on-one family support or group participation led by a trained family peer support worker [35,36].

In one feasibility study that involved reviewing medical records of patient encounters, family peer support integrated within the medical team for parents of children with special healthcare needs has been shown to enhance the care team’s ability to provide both tangible and emotional support for families [34]. In several studies, including one randomized control trial with Black and Hispanic caregivers of children with autism spectrum disorder, increases in parental knowledge growth and confidence, as well as decreased feelings of isolation and stigmatization, were observed following engagement with family peer support programs [35,37]. Qualitative research suggests that emotional support and improved feelings of resiliency from family peer support services improve caregiver reports of family quality of life and parent–child relationships [38].

### 2.4. Child and Adolescent Mental Health

There is emerging interest in exploring the roles that PPs can play in enhancing pediatric mental health care services through peer support directed toward children and adolescents as well as through peer support to parents and caregivers. Research is growing on the importance of family-focused approaches to address child and adolescent mental health needs [7,39]. Parent mental health and child mental health have bidirectional effects, with challenges for each member of the family contributing to emotional strain for other family members. Parenting a child with mental health concerns is stressful, and the parental/caregiver burden is a substantial contributor to caregivers seeking clinical services [40]. Additionally, addressing parent mental health needs and building parenting skills benefits children. While these opportunities to address family-based mental health factors are recognized, the shortage of mental health professionals, particularly acute in underserved communities, continues to limit access to clinical care. Furthermore, clinical care that is available may be lacking in cultural humility and compatibility, limiting its effectiveness and acceptability for the communities being served. In the context of these convergent factors, there is growing interest in the roles of PPs in providing targeted interventions, including through parent-to-parent peer support as well as peer mentorship to children and adolescents. Parent peers provide social support, parenting guidance, and practical assistance in navigating child-serving systems focused on mental health, education, juvenile justice, child welfare, and substance use treatment [41].

#### 2.4.1. Parent-to-Parent Peer Paraprofessional Support

A crucial and often overlooked contributor to child and adolescent mental health problems is untreated mental health problems in parents and caregivers [7]. Parent mental health difficulties contribute to child and adolescent mental health difficulties through a range of mechanisms including modeling and impaired parenting skills. Addressing symptoms in parents can have an important positive effect on offspring mental health [42]. Peer-delivered interventions offer a novel and promising strategy to address this challenge. Trained peers can help parents to identify their own mental health problems, offer psychoeducation and social support, encourage help-seeking, and reduce barriers to accessing care. Peer support provides empathetic support in addition to expressions of concern and instrumental assistance [43]. Peers can also provide scaffolding for adherence to prescribed clinical interventions by encouraging compliance with treatment and helping parents to practice behavioral strategies. Narrative reviews of peer support programs for parental mental health have found the benefits of peer support in supporting coping needs and desires for social connection and understanding from those who have experienced the mental health challenges that can accompany parenthood [43].

PPs also provide support to parents and caregivers in social situations that can challenge effective parenting. For example, peer support programs for non-resident fathers show improvements in co-parenting relationships and increased time spent with their children as a result [44]. Similarly, a program facilitating parenting skills for incarcerated mothers utilized peer support as a way for mothers in the group to provide mutual support for one another [45]. Despite preliminary evidence that supports the value of peers and community health workers to promote resilient family functioning, there are limited peer-reviewed studies in the literature.

Parent-focused and dyadic interventions that enhance parents’ capacities to support young children’s acquisition of self-regulatory, social, and cognitive skills are effective strategies for improving early childhood mental health [39]. Use of positive parenting strategies is associated with improved parent–child relationships, improved self-regulatory skills, and reduced disruptive behaviors in young children [46,47]. Positive parenting skills programs have been identified as deliverable by different interventionists, including clinicians and paraprofessionals [48,49]. Attachment-focused interventions are a second promising practice in this domain and have been developed for high-risk families who have experienced significant trauma or adversity [50,51,52]. Emerging research suggests that these interventions can be delivered by non-clinicians with appropriate training and support [53].

#### 2.4.2. Child and Adolescent Peer Paraprofessional Support

Middle childhood and adolescence are developmental stages in which children and youth build important relationships with adults outside of their family and also begin to prioritize relationships with peers [54]. Mentorship is a well-established approach to leveraging these relationships to effectively support academic success, promote healthy social and emotional development, and mitigate risk for youth [55]. Youth mentor, community mentor, health mentor, and therapeutic mentor are all examples of terms that can be used to describe an individual who provides guidance, support, encouragement, attention, and care to someone in need over an extended period of time [56]. This individual often has experience and cultural knowledge relevant to the mentee and is able to build a trusting relationship based on social, community, or school-based networks [57].

Capitalizing on the knowledge, lived experience, and cultural affinity of trained mentors offers opportunities to enhance culturally congruent and accessible mental health support [58,59,60]. For young people with mental health challenges, support from trained mentors can be an important resource for social support, modeling of positive behaviors, and scaffolding to practice skills to enhance social and emotional functioning. As such, mentors can be considered part of the peer paraprofessional workforce, provided that they receive specialized training to work with youth with mental health concerns. These trainings help mentors to develop skills including reducing stigma around seeking clinical support, connecting mentees to appropriate clinical services, and helping mentees practice mental health skills in real life outside of clinical settings.

Therapeutic mentorship is a specific paraprofessional care model with evidence of medium-to-large effect size [61]. Therapeutic mentors are trained in an array of basic therapeutic skills and work closely with licensed clinicians and the child’s family to practice emotional and behavioral skills relevant to the child’s mental health difficulties [62]. A recent publication provides a very helpful framework for integrating peer paraprofessional mentorship into the landscape of mental health services [60].

Near-age or cross-age peers are another potential addition to the peer paraprofessional workforce for supporting child and adolescent mental health [60]. A recent meta-analysis found that cross-age peer mentorship has a medium-sized overall effect on positive outcomes for youth and was particularly effective in programs in which peer mentors had strong oversight and supervision from adults [63]. Research suggests that receiving mental health support from peers is acceptable and effective for adolescents [57,64]. Improved social emotional functioning and decreased family conflict is also reported by adolescents who receive peer support mental health services [65]. Mentors who receive specific training that focuses on helping youth to develop relevant social, emotional, and cognitive skills can have a significantly positive effect on youth mental health [55,66]. Participation in mentorship programs may be more acceptable and accessible for many families who have low rates of participation in clinical mental health treatment for children and adolescents [67].

An additional role for trained mentors may be found in their deployment to augment digital mental health interventions (DHMI). DHMI are increasingly prevalent and are a promising strategy to address gaps in mental health care. A recent meta-review suggests that trained mentors may be specifically helpful in increasing the effectiveness of DHMI by providing “human support” to increase the uptake and practice of emotional and behavioral skills introduced through these digital tools [68].

## 3. The Promise of Peer Paraprofessionals for Promoting Mental Health Equity

Despite the high prevalence of mental health problems in pediatric populations and the development of effective interventions, utilization of and engagement with services remain low, with 80% of children in need of services not receiving them [69]. Rates of service access are even lower among African American and Latino families, likely due to underdiagnosis in many cases [69,70]. For some groups, such as African Americans, research suggests common usage of informal support for mental health, although research is mixed on whether these forms of support are used in lieu of or in tandem with professional mental health care [71]. Similarly, stigma related to mental health prevents individuals from seeking mental health services and research suggests that minority racial and ethnic groups are more likely to experience mental health stigma than their white counterparts [72,73]. Beyond the cultural factors and stigma that may lead individuals to prefer informal support, many people from marginalized groups have a historically-grounded mistrust of the medical system, with ongoing systemic racism further preventing access for those who seek treatment [74,75]. Even when families initiate mental health treatment, barriers to accessing basic social service needs, including lack of transportation and housing insecurity, are likely to affect mental health service utilization for low income and underserved families [76].

Incorporating PPs into family mental health services is a promising strategy to facilitate access and retention because peers are uniquely suited to enhance intervention acceptability and to address common barriers by establishing trusting relationships with families and connecting them to appropriate resources. The scope of services offered by PPs is great and includes the promotion of positive attitudes and beliefs through psychoeducation and emotional support (e.g., empowering families, stigma reduction, reducing families’ sense of isolation and self-blame), motivation enhancement through motivational interviewing techniques (e.g., empathic listening, enhancing parent self-efficacy), and addressing environmental obstacles through instrumental support (e.g., coordination of care, assistance with resources such as respite and transportation) [77,78,79]. Peer support interventions have been effective across racial/ethnic groups and health diagnoses (see [80,81,82] for examples). For groups such as refugees and immigrants who are likely to face financial, insurance, or language barriers to receiving care, peer support has been demonstrated to be culturally acceptable and to facilitate mental wellness [83,84]. The alignment of their personal background, race/ethnicity, and lived experience with that of their client’s helps peer workers to combat commonly faced barriers to mental health care for underserved families.

The use of PPs as members of the mental health service workforce may hold additional promise in addressing mental health inequities because the services that they provide are potentially easier to scale and of lower-cost compared to treatment by a licensed mental health professional. Considerations for the scalability of an intervention include its effectiveness across target groups, resources required by the workforce or organizations, acceptability of the intervention, and its compatibility with already existing programs, as well as several other factors [85]. Peer support can function as a standalone intervention and can also be integrated within existing programs to supplement professional mental health care [86]. Several family-focused interventions have documented the positive effects of peer-delivered interventions, although additional evaluation is needed [87]. Peer support can be used as a health promotion or prevention strategy in the context of a continuum of care so that families with emergent or subclinical needs are supported without needing to navigate clinical services, allowing those with greater technical skill to serve families with the highest clinical needs. When peer support is used this way, it can alleviate strain on the current mental health system.

For many individuals living with mental health conditions or facing other challenges, peer support is identified as a desired method of receiving care and support [88]. Peer support offers a recovery-focused approach that is possible due to the relationship formed between a peer worker and their client, eliminating some of the barriers that exist in the medical professional–patient dynamic [89]. The nature of the peer support–client relationship allows for increased nuance in the support workers’ understanding of the client’s challenges and needs [21,27,28]. In this way, peer support allows for the dimensionality that exists in each individual’s unique experience of mental illness that moves beyond traditional diagnosis-driven treatment approaches that may not be as effective for improving mental health outcomes [90]. Peer-delivered interventions can be tailored for specific population groups, and evidence suggests that these approaches may be more acceptable in cultural contexts in which clinical mental health care may be less desirable based upon stigma and other cultural–historical factors [21,27,28].

PP-delivered services for child, adolescent, and family mental health show promise based upon evidence from other PSW and CHW interventions in other populations, and also based upon the potential for these interventions to bridge gaps that tend to exacerbate disparities in mental health care. How can this promise be translated into additional action? The purpose of the two sections that follow is to (a) summarize existing efforts to train, deploy, and sustain PP workforces; (b) describe a new training initiative that specifically focuses on preparing PP to support infant, early childhood, and family mental health through peer-delivered support; and (c) to present new research findings from a landscape analysis in the District of Columbia that reviews current PP services available to support families.

## 4. Developing a Peer Paraprofessional Workforce

### 4.1. Training the Workforce

In order to gain the competencies necessary to provide high quality and effective services as a PP, individuals need training and experience that build their competencies across a wide array of domains. Education and training requirements for PSWs and CHWs vary across states and organizations. Although many states have adopted the APHA’s definition of a CHW and the National Association of Community Health Workers recently developed a Six Pillars of Community Health Workers definition of this role, there are currently no nationally recognized standards for CHW education or certification. Many states have professional networks and associations that provide membership and training opportunities to CHWs. The National Council for Mental Wellbeing offers behavioral health training for CHWs to help teach the necessary expertise to provide community-level behavioral health support. The training is a one-day in-person group training designed to provide CHWs with the skills and expertise needed to support people with physical and behavioral health disorders [91].

Healthy Start, a national program sponsored by the Health Resources and Services Administration (HRSA) within the Department of Health and Human Services focuses on promoting maternal and child health and reducing disproportionate rates of infant mortality across racial/ethnic groups. Given the extensive use of CHWs in their program, Healthy Start developed and published a set of core competencies for the CHWs delivering that program [92]. In addition to competence in the Healthy Start program and perinatal health, CHWs delivering the program are expected to exhibit competence in several areas related to their role including outreach, participant screening and community assessment, health education, care coordination, patient empowerment, and community engagement. Foundational competencies for Healthy Start CHWs are defined as effective communication, cultural competence, mediation, and public health.

The National Association of Peer Supporters (NAPS) is one organization that specifically develops training and credentialing standards and certification exams for PSWs. This allows organizations to utilize this credentialing service within their peer support role specifications and requirements. For instance, the Veterans Health Administration and Medicaid reimbursement for peer support services requires state peer support workers to pass the NAPS exam as a condition of hire [24]. According to a landscape report conducted by the Behavioral Health Workforce Research Center in 2019, every state besides South Dakota and Vermont offered at least one statewide peer support credential in adult mental and behavioral health, to total 65 different credential entities across the entire country. Many states allow these credentials to be transferred if a PSW moves across state lines and wants to continue their work.

The Substance Abuse and Mental Health Services Administration (SAMHSA) has a major influence on training and promoting PSWs within behavioral health settings. It develops training materials and resources for PSWs in mental health and substance recovery organizations. SAMHSA defines twelve core competencies for PSWs in Behavioral Health Services [18]. Several states offer content-specific training depending on the interest and placement of PSWs in their service systems. Many utilize the PSW training curriculum from Appalachian Consulting Group for mental health peer workers, which consists of 24 one-hour training sessions covering topics such as ethics, problem solving, and PSW wellbeing [93].

Most states also require some sort of lived experience with a behavioral health disorder to qualify for the role of PSW [94]. PSWs focused on family and child mental health may not need direct experience with the behavioral health system to be eligible for training and credentialing as a PSW. Instead, lived experience as a parent or caregiver may be more valuable and relevant. Typically, lived and practical experience are more significant credentials for PSWs than academic training. A PSW credential does not often require more than a GED or high school diploma but does often require at least 50 h of specialized training, 500 practice hours, and 25–50 supervisory hours, depending on the state [95]. A key consideration in PSW training and health system integration is also ensuring the curriculum and job duties allow for the maintenance of PSW wellbeing and self-care [96,97].

While there is much overlap in CHW and PSW skills and training, the PSW training requires lived experience, making it distinctly unique from CHW training. Additionally, the training modules for certification for CHWs are often longer than that of PSWs. Despite these differences, CHWs and PSWs addressing mental health share a common goal: bettering the mental health of at-risk populations through tailored and culturally competent approaches. In this respect, the two would benefit from interdisciplinary work, building off each other’s skills and experiences and ultimately strengthening the workforce.

### 4.2. Sustaining the Workforce

Despite the many social and economic benefits of PSWs, many states have failed to establish a sustainable PP workforce and are therefore underutilizing the potential of this service [98]. Barriers to integration of PSWs into the healthcare system include lack of formal financing to sustain the workforce, stigma from the current healthcare workforce, and policies that limit PSW’s creativity and flexibility in the services they are providing [99,100]. Policymakers that have successfully integrated PPs into the healthcare system ensure that training, credentialing, and financing structures for these workers are equivalent to the economic value that they bring.

Sustainable financing is one of the most important aspects of integrating PPs into a statewide healthcare infrastructure. As of 2019, 39 states included PSWs in their Medicaid billing schedules [94]. While there is no statistically significant correlation between Medicaid funding and availability of PSWs, a stable payment structure such as this promotes work–life balance and reduced burnout which contribute to a more sustainable workforce [96]. As of 2019, 33 states had credentialing for parent and or youth peer support that could be billed through Medicaid, though the specific definition of that credentialing and the billing code for peer support varied by state [101]. This report documents that most of these states utilize their state Medicaid plan as the funding authority, but some use other means of fundings including grants and institutional funds.

Of the many approaches states have taken to integrate PSWs into their healthcare system, the most effective policies have reduced stigma, ensured financial stability, and allowed for a standardized but flexible training curriculum. States hoping to expand and sustain their PSW workforce should ensure that PSWs are provided with the tools and resources to do their job and should enforce policies that promote the dignity and respect for the importance of peer support in mental health treatment settings.

### 4.3. Family Leadership Track of the Georgetown IECMH Certificate Program

The Early Childhood Innovation Network (ECIN) at Georgetown University (GU) has developed an innovative training program for PPs through the Family Leadership Track of its Infant and Early Childhood Mental Health (IECMH) Certificate Program. The Family Leadership Track (FLT) builds off of the core competencies established in the PSW and CHW literature to provide an opportunity for caregivers to develop the core competencies of a CHW with knowledge specialization in infant, early childhood, and family mental health and learn to harness the power of their lived experiences to support other caregivers. The IECMH certificate program prepares participants to work in their own communities to promote resilience in families by connecting parents, children, and caregivers to resources and fostering family strengths with high quality practices in IECMH prevention, promotion, and support. The certificate is offered through the Georgetown University School of Continuing Studies (GU SCS) which has an extensive history of providing a variety of professional certificates across education, health, and policy. Recruitment efforts were supported through posting on the GU SCS website. Additionally, the developers of the FLT leveraged ECIN’s collaboration with the Georgetown University Center for Child and Human Development (GUCCHD). For more than four decades, the GUCCHD has been recognized as a national, regional, and local leader in the field of child development. GUCCHD houses and leads several national centers that are focused on supporting the development of Early Childhood Education systems and the concomitant workforce. Similarly, ECIN’s extensive collaboration with local community organizations provided a gateway for attracting participants.

The FLT is unique because it is the only certificate program at GU SCS that focuses on increasing educational and workforce development opportunities for first-generation students who have a GED or high school diploma. Beyond first-generation students, the educational space is blended with educators, family engagement specialists, social workers, and infrastructure builders from diverse professional settings. The course is grounded in a commitment to health equity and social justice. It is designed to increase the competence of individuals historically impacted by structural racism who live in medically underserved and economically underdeveloped communities and to build and diversify the mental health workforce. The program prioritizes the recruitment of historically marginalized caregivers of young children as participants. The definition of “lived experience” for these participants is broader than how it is typically defined in peer support programs that focus on mental health or substance use recovery. The first cohort of certificate participants were recruited in 2021. There were 21 applicants for the program and 14 enrolled in the course. All fourteen students completed all requirements of the certificate program and graduated from the program. The majority of students in the program were African American. A little more than a quarter had a High School Diploma or GED, a third had a Bachelor’s Degree, two students had a Master’s Degree, and a few were currently enrolled in a degree program. More than half of the students reported having some type of specialty training or professional certification. Of these students, the majority had prior peer or family support training. However, of the students with prior training, very few reported that they had received any prior training in mental health.

#### Next Steps in the FLT of the IECMH Certificate Program

In response to feedback from the participants in cohort one, the course was updated in year two to include a focus on all CHW competencies and an apprenticeship opportunity. The current course is offered virtually, organized into 10 consecutive months of modules that focus on PP and IECMH competencies, and involves both synchronous and asynchronous learning. Over the ten months of the program, coursework is divided into twelve modules which focus on public health principles, the social determinants of health, personal wellness, attachment and social-emotional wellbeing of young children, motivational interviewing, basics of care coordination and system navigation, trauma, community asset building, advocacy, case documentation, and foundations of adult learning. Beyond the modules, the certificate program incorporates a 200-h competency-based practicum, weekly one-on-one professional success coaching, weekly facilitated group peer support sessions and mindfulness classes, monthly one-on-one wellbeing mentorship with a clinical psychologist that includes development and implementation of personal wellbeing plans, and professional training (e.g., resume creation, interview skills, and career navigation supports). Students enrolled in the program experiencing underemployment have the opportunity to participate in apprenticeships in healthcare, early childhood education, and community-based settings, and are paid stipends as apprentices.

## 5. Current Study: The Landscape of Peer Paraprofessionals for Families with Young Children in DC

In order to further refine the FLT of the IECMH Certificate Program and understand how to maximize its successful dissemination, members of ECIN’s research and policy teams designed a qualitative study of the peer support services available to families with young children in DC. Our approach was to conduct semi-structured interviews with a convenience sample of agency and organization leaders that serve families with young children.

### 5.1. Methods

Eighteen semi-structured interviews were conducted with 19 leaders from 11 organizations serving families with young children in Washington, DC. Participating organizations represented the health sector, early childhood education, mental health, and community-based organizations. Leaders represented 18 programs across these organizations. We use the term program to refer to a defined team of individuals who serve a defined group of recipients. Interviews ranged in duration from 20 min to 1 h and 29 min and lasted an average of 38 min. Interviews were conducted and recorded via Zoom and were professionally transcribed. Participants received a USD 75 gift card for completing the interview. Transcripts were managed and coded using Dedoose (SocioCultural Research Consultants, LLC, Los Angeles, CA, USA), a mixed methods research software program [102]. The research team reviewed the transcripts, noted themes, and came together to discuss the development of the codebook. After the initial codebook was developed, two interviews were coded by two separate members of the research team and the team of four coders came together to review discrepancies and reach consensus. After the initial round of coding and discussion, the codebook was revised and finalized. The remaining 16 transcripts were fully coded by one coder and 20% of each transcript was coded by a second person. The double coded excerpts were reviewed and discussed by all four coders to reach agreement. The Institutional Review Board at the lead author’s home institution determined this study to be exempt.

### 5.2. Qualitative Results

Table 1 provides a comprehensive overview of key themes present in the analysis of the interviews and selected quotes to illustrate each theme.

Type of Support Provided. The programs discussed during the interviews provided peer paraprofessional support services to a variety of people. All programs included in the analysis provided services to families with young children, but not all programs were developed specifically for this population. Conversations with participants revealed that family support workers often provide a broad range of support services for their clients. The supports described by program leaders included, but were not limited to, conducting outreach and recruitment, encouraging client engagement, providing information on available resources, making referrals, and assisting with system navigation, running educational or support groups, and empowering caregivers to advocate for themselves and their children. Some participants discussed the level of variation in the role of a peer professional as a challenge within the field.

Characteristics of the Workforce. The titles that programs use to describe the individuals on staff who provide support to families varied by program. Of those discussed, seven included the word “family”, five included the words “support worker”, four included the word “peer”, and three included the word “specialist”. The majority of positions were full-time, benefit-eligible positions. However, some positions were part-time or paid hourly as needed. Although opinions regarding the necessity of a degree varied, participants consistently described the value of lived experience for facilitating connections between the peer paraprofessional and the family.

Training and Supervision. Although few respondents mentioned requiring specific training or certification of applicants for these positions, nearly all participants in the study with active peer support programs mentioned providing several ongoing training opportunities for the peer support workers after they were hired onto the program. Training opportunities included things such as motivational interviewing, setting boundaries, resource navigation, Mental Health First Aid, and protective factors training.

Organizational Challenges. Participants discussed several challenges faced by their organizations when incorporating peer paraprofessionals into their programming including capacity for recruitment and supervision, sustainable funding mechanisms, and retention.

System Level Infrastructure. Participants were specifically asked to think about professional or governmental support that would contribute to the development of the PP workforce. The establishment of professional standards emerged as a theme during our coding. Participants emphasized that PPs should be viewed as a professional role and suggested that local government or professional agency standards could contribute to affirming the professional role of peers. Establishing evidence for PPs was discussed as one way to contribute to the legitimacy of the role.

## 6. Discussion and Next Steps for Peer Paraprofessional Models for Child, Adolescent, and Family Mental Health

Growing evidence demonstrates the effectiveness of peer support for improving outcomes across a range of physical and mental health concerns, including family mental health. Expanding the PP workforce to specialize in child, adolescent, and family mental health has the potential to address increasing demands on an already strained healthcare system. While the extant literature provides preliminary evidence that peer support can address family mental health concerns and be integrated with professional mental health services, additional research is needed to identify the core components of effective peer support for families, the mechanisms driving its effectiveness, and how to implement it in service systems to maximize its benefits for children, adolescents, and the adults that care for them.

Our literature review suggested that the educational and training requirements for PPs varied across states and organizations and whether the provider was considered a CHW or a PSW. PSWs have nationally recognized training, credentialing standards, and certification exams in most states, whereas certificate programs at the state level are typically used to provide training and field experience in public health to CHWs. The Family Leadership Track of our GU IECMH Certificate Program has evolved over the past year to become a training for community mental health workers (CHWs) who promote infant, early childhood, and family mental health.

The qualitative study presented in this paper was conducted so that we could better understand the availability of peer paraprofessional support for families with young children in the District of Columbia, which would allow us to further refine the certificate program and understand how to disseminate it in the city. Our findings were consistent with the broader research literature in terms of documenting that PPs working in our community provide a broad range of support services for their clients including access to resources, system navigation, fostering engagement in program, intervention delivery, and empowering caregivers to advocate for themselves and their children. One of the most consistent findings was the perceived value of PPs by the participants, and while organizations varied in the extent to which they required their PPs to have specific experiences to be eligible for this role, most agreed that “lived experience” facilitated a connection between PPs and the individuals they serve. However, many cautioned that attempts to standardize this concept must allow for appropriate variation in its definition and application.

Respondents in our study did not require any training or certification for the PPs yet several participants indicated that there is a need for this type of workforce development program in DC. In the context of our Certificate Program, we have learned that the cost of peer paraprofessional training programs can hinder students from enrolling and impact whether they can participate for the duration of a program. Providing funding for students to attend a training program such as the one created at GU SCS increases the educational and workforce opportunities for individuals with lived experience to leverage their experiences on behalf of others. We are confident that this certificate program will prepare them for successful peer-led positions within the communities they serve and realize that we will need to make program leaders in DC aware of what graduates of FLT have to offer. We intend to use the next several years to document the effects of PPs on family mental health outcomes and advocate for sustainable funding to support this work locally. We also hope to facilitate a network of family-focused PPs to continue to learn from those who deliver and receive peer support.

### Limitations

There were limitations to our study that should be acknowledged. Interviews highlighted in this analysis may not be representative of all family-serving organizations in the city. Although there are countless family-serving organizations within the Washington, DC Metro area, only nineteen were available to participate due to time and resource constraints surrounding the study interview process. Second, the study was a limited convenience sample of agency and organization leaders in the Washington, DC area; therefore, it may not be generalizable outside of DC. Despite these limitations, our interviews were a rich source of information that will be useful for developing additional research to better understand PP services in DC and other cities across the country.

## 7. Conclusions and Future Directions

One challenge to expanding research is the fact that many PP programs are developed within community organizations where evidence-informed practice may be more feasible than evidence-based practice [103,104]. Evidence-informed practice is often more flexible and able to adjust to the unique and dynamic needs of specific populations [105]. An important next step for the literature on PP programs includes increasing the dissemination of evidence-informed programs that are benefiting diverse groups and are easily scalable so that these models have the potential to inform what is being evaluated and considered for large-scale dissemination. Many PP innovations that improve access to quality mental health care are being implemented and evaluated outside of the peer-reviewed literature. Without continuing to consider data from a variety of sources, and without developing research–practice partnerships that value co-creation, it is likely that we are missing invaluable insights.

Early childhood is a period of foundational development heavily dependent on the caregiving environment, making it an age that may particularly benefit from peer support services for the family. However, including middle-childhood through adolescence in paraprofessional mental health certificate programs is a future direction that should be emphasized in the field for its potential to support improved mental health outcomes across the course of life. Expanded training opportunities for therapeutic mentorship and other PP models that directly support children and adolescents will accelerate the dissemination of this effective mental health strategy. Untreated mental health conditions result in significant costs to society through a range of direct and indirect economic impacts, and most mental health conditions begin in childhood and implicate the health of entire families [106]. PP models are very promising approaches to enhancing the responsiveness, reach, and cost effectiveness of systems seeking to address these challenges.

## Figures and Tables

**Table 1:** Results of thematic analysis from semi-structured interviews.

Overview of Current Family Support Programming in DC
Types of Support Provided
*“... sitting down with [the client] and having the knowledge and the connections and the ability to navigate and just to be able to fill those gaps and then just starting at the top of that list [of needs] and moving down”.*
*“This idea that families are not cases, and so it’s not just about connecting poor people to programs that poor people need, like social work. There’s some element of developing and helping a family to cultivate its own agency”.*
**Characteristics of the Support Workforce**
Education and Experience
*“It’s really more of a community experience. It’s work experience in addition to the life experience. We do have those who are college educated, but some that do not have degrees”.*
*“I’mma be honest. In reference to having a degree, I say no. I just say the experience means more because if you’ve been in a situation and you know how it feels to be without, or you’re a single mom, and you just wanna show moms or dads or caregivers where to get resources from, you don’t have to have a degree. You just gotta have passion and you gotta wanna do this work”.*
**Characteristics of Programs Utilizing Support Workers**
Organizational Challenges
*“I think there’s a lot of non-billable time that’s a part of supporting that role, so it’s not just one person that we would hire. I think, in the past, what we’ve seen when we’ve incorporated peer support [workers] is that they also need a lot of support, so you need other people to be involved to make sure that they are getting the scaffolding they need in the role”.*
*“It’s not a position that, right now, you can bill for, so that makes it hard to be sustainable. I think funding is a big thing”.*
**System Level Infrastructure for the Support Workforce**
Establishing Professional Standards
*“If I were gonna launch a true peer navigator and not have a bachelor’s degree requirement and be pulling in people from a really diverse depth of professional experience or something. Some who may have done a lot professional work and some who maybe have done very little, but have this really great lived experience, then I would imagine that if the city could provide some kind of structured modules of these are the basics of peer support and confidentiality, and I don’t know. Just those types of things”.*
Developing the Profession
*“It may be helpful to have a peer-to-peer parity program… it’d be nice for all of them to get together and talk about the strengths, the challenges, and some of the struggles associated with it. I think that another area that will be very helpful is for them to process the trauma that is induced by the work. If I had experienced what you’re experiencing, then I can be triggered by supporting you. Some place that captures some of that and help them with managing that”.*

## Data Availability

Data are unavailable due to privacy restrictions.
